# Genetic Variations in the Serotonergic System Mediate a Combined, Weakened Response to SSRI Treatment: A Proposed Model^1^,^2^

**DOI:** 10.1523/ENEURO.0032-14.2015

**Published:** 2015-06-05

**Authors:** Adam Pettitt

**Affiliations:** 1 Department of Psychology, University of Oregon, Eugene, Oregon 97403; 2 Department of Biology, Western Oregon University, Monmouth, Oregon 97361; 3 Department of Psychology, Western Oregon University, Monmouth, Oregon 97361

**Keywords:** 5-HT1A, 5-HTTLPR, depression, psychopharmacology, serotonin, SSRI

## Abstract

Individuals with the short (S) allele in the promoter region of the serotonin transporter gene (5-HTTLPR) show a less favorable response to selective serotonin reuptake inhibitor (SSRI) treatment than individuals with the long (L) allele. Similarly, individuals with the C(-1019)G allele for the mutation found in the promoter region of the serotonin 1A receptor gene (5-HTR1A) have shown blunted responses to SSRI treatment when compared with individuals lacking this polymorphism. While these findings have been replicated across multiple studies, only two studies to date have reported data for a gene–gene interaction associated with response to SSRI treatment. Both of these studies reported a combined effect for these genotypes, with individuals homozygous for the L allele and the C allele (5-HTT^L/L^−1A^C/C^) reporting the most favorable response to SSRI treatment, and individuals homozygous for the S allele and the G allele (5-HTT^S/S^–1A^G/G^) reporting the least favorable response to SSRI treatment. Additionally, no neural mechanisms have been proposed to explain why this gene–gene interaction has been observed. To that end, this article provides a review of the relevant literature associated with these polymorphisms and proposes a feasible model that describes a genotype-dependent modulation of postsynaptic serotonin signaling associated with the 5-HTT and 5-HTR1A genes.

## Abstract (precis)

Approximately forty percent of individuals that seek pharmacological treatment for depression do not initially respond to selective serotonin reuptake inhibitor (SSRI) antidepressants. Past research has attempted to determine if specific mutations in genes associated with the serotonergic system can help to predict response to antidepressant treatment; however, results have been inconclusive.

## Significance Statement

Approximately forty percent of individuals that seek pharmacological treatment for depression do not initially respond to selective serotonin reuptake inhibitor (SSRI) antidepressants. Past research has attempted to determine if specific mutations in genes associated with the serotonergic system can help to predict response to antidepressant treatment; however, results have been inconclusive. Additionally, very little research has examined how multiple mutations can cause a combined, reduced response to SSRI antidepressant treatment. This article provides a review of the relevant literature, offers a model for why individuals with multiple mutations in the serotonergic system show a blunted response to SSRIs, and provides a basis for further research regarding genotype-dependent response to antidepressant treatment.

## Introduction

Major depressive disorder (MDD) is a mental disorder that will affect an estimated 16% of the world population (Kessler et al., 2003). Of those individuals who seek treatment, approximately one-third of patients do not respond to antidepressant therapies (Fava and Davidson, 1996; Fava, 2003; Papakostas et al., 2006; Trivedi et al., 2006). Due to the high prevalence of MDD, and its associated healthcare costs, there has been a dramatic increase in the amount of money spent on both the prevention and treatment of this disease (Wang et al., 2003; Halfin, 2007). Because of these expenses, researchers have sought to better tailor treatments to individuals in hopes of reducing the vast resources expended pursuing effective treatment options. One proposal for reducing overall treatment cost is via genetic testing, which could help to predetermine which individuals will favorably respond to specific treatment types (Rausch et al., 2002). Unfortunately, further knowledge in regard to the underlying mechanisms by which individual genotypes are expected to interact with various drugs is necessary before genetic-testing techniques can be implemented (Serretti et al., 2009). Currently, much of the research being conducted on depression focuses on a class of signaling molecules known as neurotransmitters.

Reduced amounts of the monoamine neurotransmitter serotonin [5-hydroxytryptamine (5-HT)] is thought to be a predisposing factor for susceptibility to depression (Murphy et al., 1998). Additionally, short-term depletion of tryptophan, a biochemical precursor for 5-HT, leads to decreased serotonin levels, which can mediate relapses in previously depressed patients (Booij and Van der Does, 2011; Yatham et al., 2012; Young, 2013). In the brain, serotonin is produced by a subset of neurons located in the raphe nuclei (RN); however, axons extending from the RN innervate large areas of the brain, including the prefrontal cortex, hippocampus, hypothalamus, and amygdala (Peyron et al., 1998; Hornung, 2003). These serotonergic projections modulate a multitude of behavioral responses, including sleep circadian rhythms, satiety levels, and mood (Bauer et al., 2002; Kranz et al., 2010; Homberg and Lesch, 2011); disruptions within these pathways have been linked to depressive symptoms (Holmes, 2008; Albert et al., 2014).

While serotonin has been implicated as an important biological factor in depression, many other factors, such as environmental stress and genetic makeup, can contribute to susceptibility to depression (Pittenger and Duman, 2008; Jasinska et al., 2012). The most widely studied mutation in the serotonin system is a variation in the length of the promoter region, known as the promoter region of the serotonin transporter protein (5-HTTLPR), located upstream of the serotonin transporter gene (SLC6A4; Murphy et al., 2004). Varying numbers of repeated elements are associated with different alleles; the “long” allele (5-HTT^L^) has 16 repeat elements, while the “short” allele (5-HTT^S^) has 14 repeat elements. The 5-HTT^L^ allele is associated with a twofold increase in the basal serotonin transporter protein (5-HTT) transcription rate when compared with the 5-HTT^S^ allele, due to increased transcription factor binding of the 5-HTT promoter region (Maier and Zobel, 2008).

The presence of the 5-HTT^S^ allele has been associated with an increased susceptibility to depression in multiple studies. Interestingly, this increased susceptibility to depression exists only when preceded by stressful life events, suggesting that susceptibility to depression is mediated by both genetic and environmental factors (Caspi et al., 2003; Risch et al., 2009; Goldman et al., 2010; Uher and McGuffin, 2010; Karg et al., 2011). As well as being linked with increased susceptibility to depression, individuals with the 5-HTT^S^ allele in the 5-HTTLPR have also been shown to be less responsive to selective serotonin reuptake inhibitor (SSRI) treatment than individuals with the 5-HTT^L^ allele (Smits et al., 2004; Porcelli et al., 2012).

In addition to the 5-HTTLPR polymorphism, a single nucleotide polymorphism in the promoter region of the serotonin 1A autoreceptor (5-HT1A) serotonin receptor gene (5-HTR1A) is also associated with depressive phenotypes. The G allele associated with this polymorphism, also known as C(-1019)G or rs6296, has been linked to a blunted response to SSRI treatment (Parsey et al., 2006b, 2010). As both of these polymorphisms are located within the promoter regions of each gene, the transcription rate of both the 5-HTT and the 5-HT1A receptor is altered when these alleles are present (5-HT^S^ and 5-HT1A^G^, respectively). These altered transcription rates lead to altered transporter and receptor expression, and are a possible explanation for why these polymorphisms cause blunted responses to SSRI antidepressant therapy.

While many studies have examined the association between one polymorphism genotype and the response to SSRI treatment, only two studies to date (Arias et al., 2005; Hong et al., 2006) have accounted for a gene–gene interaction associated with treatment response. These studies showed a combined genotype effect for SSRI response, suggesting that some of the discrepancies found in other studies (Kraft et al., 2007; Dogan et al., 2008; Wilkie et al., 2008), which focused solely on one polymorphism, could be attributed to the omission of other genotypes. While there was a gene–gene interaction of 5-HT1A and 5-HTTLPR polymorphisms reported in both studies (Arias et al., 2005; Hong et al., 2006), no underlying mechanism has been offered as an explanation for why these findings were observed.

The purpose of this review is to provide a background on studies relevant to these polymorphisms and to propose a neural model that could account for the combined effect noted in these two studies. The neural model offered is based on alterations in the transcriptional regulation of the 5-HTT and the 5-HT1A receptor genes, with the 5-HTT^S^ allele causing a reduction in transcription rate of the 5-HTT and the 1A^G^ allele causing an increase in the transcription rate of the 5-HT1A receptor. Ultimately, the model hypothesizes how these altered transcription rates affect the amount of extracellular serotonin available for postsynaptic signaling and predicts a genotype-dependent response to SSRI antidepressant treatment.

## Serotonin transporter protein

5-HTT, which is encoded by the human SLC6A4 gene, is thought to be the main mechanism by which serotonin is removed from the synapse (Homberg et al., 2007; Murphy and Lesch, 2008). While some mutations in the SLC6A4 gene have been shown to cause significant impairments in response to SSRI treatment (Smits et al., 2004), the majority of studies have focused on polymorphisms within 5-HTTLPR. In addition to the previously mentioned 5-HTT^L^ and 5-HTT^S^ polymorphisms, the 5-HTTLPR can be further modified by another allele, the 5-HTT^LG^ allele (Hu et al., 2006; Murphy et al., 2013). This 5-HTT^LG^ allele is associated with decreased transcription rates that could be equivalent to the 5-HTT^S^ allele. In other words, individuals with the 5-HTT^L/L^ genotype could have a twofold increase in the expression of 5-HTT when compared to individuals with the 5-HTT^LG^ or 5-HTT^S^ allele.

This twofold increase in expressed 5-HTT increases the amount of 5-HT removed from the synapse, also by a factor of two (Lesch et al., 1996; Bengel et al., 1998; Montañez et al., 2003; Homberg et al., 2007). Perhaps paradoxically, however, 5-HTT knock-out studies have shown there are no discernible differences in extracellular 5-HT concentrations in mice with half the amount of normally expressed 5-HTT (5-HTT^+/−^) compared with mice with normal 5-HTT (5-HTT^+/+^) expression (Bengel et al., 1998; Mathews et al., 2004; Shen et al., 2004). Other studies have shown that 5-HT reuptake is also carried out by other monoamine transporters (e.g., dopamine, norepinephrine), which could compensate for the reduced uptake in 5-HTT^+/−^ mice (Shen et al., 2004). However, studies have reported significantly higher extracellular 5-HT concentrations in mice expressing no 5-HTT (5-HTT^−/−^) compared to 5-HTT^+/+^ and 5-HTT^+/−^ mice, demonstrating that other monoamine transporters account for only a minimal amount of 5-HT reuptake (Mathews et al., 2004; Shen et al., 2004; Homberg et al., 2007).

Another explanation for the similarities in 5-HT concentration in both 5-HTT^+/+^ and 5-HTT^+/−^ mice is that basal firing rates of medial RN 5-HT neurons were reduced by 36% in 5-HTT^+/−^ mice when compared to their homozygous 5-HTT^+/+^ littermates (Gobbi et al., 2001). This reduced firing rate is attributed to the negative feedback mechanism mediated by 5-HT1A receptors, which alter the amount of 5-HT being released in direct response to how much 5-HT is being removed by the transporter (Fabre et al., 2000). This effect on the signaling pathway is thought to play a large part in patient response to antidepressant treatment, which is why the most widely prescribed antidepressant pharmacological agents (SSRIs) impinge on the 5-HTT.

### 5-HTT and SSRI

SSRIs are thought to inhibit the reuptake of 5-HT by binding to the active site of the extracellular hydrophobic region of the 5-HTT, effectively reducing the uptake of extracellular 5-HT (Murphy et al., 2004). As the 5-HTT^S^ allele is associated with a reduction in the transcription rate of the 5-HTT by a factor of two, neurons with at least one 5-HTT^S^ allele express half the amount of 5-HTT when compared with neurons with the homozygous 5-HTT^L/L^ genotype. Because fewer 5-HTTs are available, studies have demonstrated that neurons expressing the 5-HTT^S^ allele take approximately double the amount of time to remove 5-HT from the synapse when compared with neurons expressing 5-HTT^L^ alleles (Lesch et al., 1996; Bengel et al., 1998; Montañez et al., 2003; Homberg et al., 2007). However, to determine 5-HT reuptake rates, the majority of these studies exogenously add 5-HT to the surrounding synapses. These results may not accurately represent the true nature of what is occurring at the synapse following SSRI treatment.

As previously stated, rodents with reduced expression of 5-HTT had no discernable differences in extracellular 5-HT prior to SSRI treatment. This, coupled with the observation that 5-HTT^+/−^ mice show reduced serotonergic firing rates (Gobbi et al., 2001), provides a possible explanation for what is occurring at the synapse after 5-HTT inhibition. Following SSRI treatment, if the same proportion of 5-HTTs are blocked in both 5-HTT^+/+^ and 5-HTT^+/−^, then less 5-HT is ultimately released into the synapse. This is due to the 5-HT^+/−^ neurons having reduced amounts of serotonin being released because of lower firing rates. Ultimately, Because less 5-HT is being released into the synapse in 5-HTT^+/−^ neurons, 5-HTT^+/+^ neurons will have a larger increase in extracellular 5-HT after 5-HTT reuptake inhibition when compared with extracellular concentrations of 5-HT in 5-HTT^+/−^ neurons. Supporting this proposed mechanism, subsequent to reuptake inhibition, 5-HTT^+/+^ mice had significantly higher extracellular 5-HT concentrations than 5-HTT^+/−^ mice (Shen et al., 2004).

These results yield a possible explanation for why depression studies have reported an association between 5-HTT^S/S^ individuals and blunted SSRI treatment response (Smeraldi et al., 1998; Serretti et al., 2007; Huezo-Diaz et al., 2009; Porcelli et al., 2012). Participants with differing genotypes receiving the same amount of SSRI should respond differently to treatment. This is mainly because individuals with the 5-HTT^L^ allele would have a higher 5-HT concentration following SSRI treatment and, subsequently, more 5-HT postsynaptic signaling than individuals with the 5-HTT^S^ allele. Corroborating this, individuals with the 5-HTT^S^ allele showed reduced response to SSRI treatment when compared with individuals with the 5-HTT^L^ allele. However, when the dosage of the SSRI was increased in individuals with the 5-HTT^S^ allele, these individuals showed favorable responses that were similar to individuals with the 5-HTT^L^ allele (Rausch et al., 2002). This increased dosage would lead to increased circulating SSRI levels. Ultimately, as more 5-HTTs are inhibited from the increased circulating levels of the SSRI, more 5-HT is kept in the synapse. This could compensate for the reduced amount of extracellular 5-HT in neurons expressing fewer 5-HTTs following SSRI treatment (Gartside et al., 1995), resulting in a reduction of depressive symptoms.

However, this initial increase in extracellular 5-HT levels does not fully explain SSRI mechanisms in their entirety; if reuptake inhibition were the only mechanism occurring, a response to SSRI treatment would happen almost instantaneously. Instead, patients report an initial response somewhere between 4 and 8 weeks after beginning treatment (Steimer et al., 2001; Gibbons et al., 2012). Many researchers implicate the downregulation of the 5-HT1A as the most probable cause for this delay in response (Trivedi et al., 2006; Catapano and Manji, 2007; Hannon and Hoyer, 2008). While past antidepressant treatment options have been generally geared toward the 5-HTT, recently developed pharmacological agents have been aimed at other aspects of the serotonergic system, such as the 5-HT1A receptor (Lladó-Pelfort et al., 2010).

## 5-HT1A

Recently, the 5-HT1A receptor has been implicated in both susceptibility to depression and as a factor moderating the response to SSRI treatment (Blier, 2010; Richardson-Jones et al., 2010, 2011; Donaldson et al., 2013). The serotonin 1A receptor is a presynaptic autoreceptor in the RN, and a postsynaptic heteroreceptor in the amygdala, cortex, hippocampus, and hypothalamus (Sotelo et al., 1990; Burnet et al., 1995; Riad et al., 2000). The serotonin 1A receptor is a pertussis toxin-sensitive heterotrimeric G-protein-coupled receptor (GPCR), which is coupled negatively to adenylyl cyclase (Catapano and Manji, 2007). Ligand binding to this class of GPCRs has been shown to open potassium channels and close calcium channels, leading to hyperpolarization of the neuron and ultimately to inhibition of the cell (Rotondo et al., 1997; Hannon and Hoyer, 2008).

Serotonin 1A receptors are the main somatodendritic autoreceptors that mediate the negative-feedback mechanism in serotonergic raphe neurons (Riad et al., 2000). Discrete portions of the RN have serotonergic axons that extend to different regions of the brain (Bang et al., 2012). Within each of these discrete clusters of neurons, shorter axons extend from the RN, synapse onto other serotonergic axons, and inhibit the axons extending to other portions of the brain (Vasudeva et al., 2011). This feedback mechanism works by inducing a series of cascade events that regulate the amount of serotonin being released based on current extracellular 5-HT concentrations. In other words, a decrease in extracellular 5-HT will result in reduced 5-HT1A autoreceptor binding, less cell hyperpolarization, disinhibition of the serotonin neuron, and increased 5-HT release at the axon terminal. Conversely, an abundance of extracellular 5-HT will result in increased 5-HT1A autoreceptor binding, increased cell hyperpolarization, neuronal inhibition, and reduced 5-HT release at the axon terminal (Koek et al., 1998; Gobbi et al., 2001).

### 5-HT1A genes

The 5-HT1A receptor is encoded by the intronless gene 5HTR1A, which is found on chromosome 5q11.2-13 (Kobilka et al., 1987; Albert et al., 1990). Similar to the 5-HTTLPR region in the 5-HTT gene, the 5HTR1A gene contains a promoter sequence. However, the interactions between the promoter region and transcription factors associated with this area are more complex (Parks and Shenk, 1996; Albert et al., 2011). Pet-1 is a transcription factor, expressed only in raphe-specific cells, that binds to several Pet-1 binding sites upstream of the 5HTR1A gene (Jacobsen et al., 2011). Because Pet-1 is only expressed in serotonergic neurons found in the raphe nuclei, all 5-HT1A receptors produced by Pet-1 are considered autoreceptors. More broadly, 5-HT1A receptors—both heteroreceptors and autoreceptors—are selectively expressed only on neuronal cells via the repressors Freud-1, Freud-2, and REST (Ou et al., 2000; Lemonde et al., 2004). While REST restricts the expression of 5-HT1A receptors only in non-neuronal cells. Freud-1/2 repressors act to restrict 5-HT1A receptor expression in both non-neuronal and neuronal cells (Albert, 2012). More specifically, Freud-1/2 repressors act to inhibit the expression of 5-HT1A receptors in the brain and the CNS.

Another transcription factor regulating the expression of 5HTR1A is NUDR/Deaf1, which is unique in that its function changes depending on which cell type it is expressed in. In non-raphe neurons, this transcription factor acts as an enhancer, which upregulates the amount of postsynaptic heteroreceptors present on the cell membrane (Czesak et al., 2006). In direct juxtaposition, the same transcription factor (Deaf1) acts as a repressor in RN serotonergic cells. A study supporting this reported a 50% increase in 5-HT1A autoreceptor expression in the dorsal and medial RN following removal of the Deaf1 repressor (Czesak et al., 2012). This relatively recent discovery has elucidated some of the mechanisms surrounding 5-HT1A receptor regulation and 5-HT1A receptor binding in depression, as multiple conflicting studies report both increased and decreased 5-HT1A binding in depressed individuals (Parsey et al., 2010).

### 5-HT1A and depression

Many of the first studies implicating 5-HT1A receptors in depression consisted of postmortem studies of depressed suicidal patients (Stockmeier et al., 1998; Arango et al., 2001). Discrepancies arose, however, as these studies reported both decreased and increased 5-HT1A binding in suicidal individuals. These studies have been criticized for their very low sample size and high interperson variability; more specifically, age varied widely among subjects in these postmortem studies. As positron emission tomography (PET) imaging of the 5-HT1A selective antagonist [^11^C]WAY-100635 has shown significant decreases in 5-HT1A receptor binding with increased age (Tauscher et al., 2001), this has cast some doubt on the reliability of these findings.

PET scanning is considered a more reliable method for determining the association of 5-HT1A binding with depression, as this technique allows for measurements of metabolic changes in live tissue (Sargent et al., 2000; Bhagwagar et al., 2004; David et al., 2005; Parsey et al., 2006a). Many of these studies reported findings that conflicted with one another, as some studies associated increased depressive symptoms with reduced 5-HT1A receptor binding (Drevets et al., 1999; Sargent et al., 2000). Others studies, however, reported that higher binding potentials were correlated with increased depressive symptoms and blunted SSRI treatment response (Parsey et al., 2006a, b, 2010; Miller et al., 2009; Richardson-Jones et al., 2010).

A possible reason for these discrepancies has been attributed to the C(-1019)G polymorphism located within an inverted repeat section recognized by the Deaf1 transcription factor (Jacobsen et al., 2011; Albert, 2012). As noted earlier, Deaf1 acts as an enhancer in non-raphe cells (increasing 5-HT1A heteroreceptor expression) and a repressor in raphe cells (decreasing 5-HT1A autoreceptors expression; Czesak et al., 2006; Czesak et al., 2012). This disruption in the expression of the 5-HT1A autoreceptor has been associated with decreased SSRI response (Serretti et al., 2004; Suzuki et al., 2004; Arias et al., 2005; Hong et al., 2006) and increased susceptibility to depression in adults directly following a stressful life event (Lemonde et al., 2003; Parsey et al., 2010; Benedetti et al., 2011). Additionally, studies have revealed that individuals with the 5-HTT^S/S^ genotype show reduced expression of the 5-HT1A autoreceptor, which could also account for an increased susceptibility to depression (David et al., 2005).

Studies using both genetic and neuroimaging techniques have found increased presynaptic 5-HT1A autoreceptor raphe binding associated with the 1A^G/G^ genotype (Parsey et al., 2006b, 2010), whereas other studies (Sargent et al., 2000; Bhagwagar et al., 2004) have reported reduced binding across postsynaptic 5-HT1A heteroreceptors. Interestingly, some studies (Parsey et al., 2006b) have reported an increase in postsynaptic 5-HT1A binding potentials associated with the 1A^G/G^ genotype. While this genotype does result in significant downregulation of the 5-HT1A heteroreceptor, increased 5-HT1A autoreceptor expression leads to decreased extracellular 5-HT concentrations. This decrease in extracellular 5-HT concentrations is thought to lead to partial, compensatory upregulation of the postsynaptic 5-HT1A heteroreceptor (Parsey et al., 2010; Albert, 2012). This compensation would account for the observed increased postsynaptic binding. In addition, increased 5-HT1A autoreceptor binding [especially due to the C(-1019)G polymorphism] has been correlated with a decreased response to SSRI treatment (Parsey et al., 2006b; Richardson-Jones et al., 2010). This blunted response in participants with the 1A^G/G^ genotype is attributed to increased raphe inhibition due to higher 5-HT1A autoreceptor binding. This increased inhibition would ultimately lead to overall lower concentrations of extracellular 5-HT when compared with neurons from individuals with the 1A^C/C^ genotype (Albert et al., 2014).

Adding to this line of evidence are a number of studies in which mice have been transgenically bred to show normal, reduced, or no 5-HT1A receptors (1A^+/+^, 1A^+/−^, 1A^−/−^ mice, respectively; Donaldson et al., 2013). In a 2010 study, researchers (Richardson-Jones et al., 2010) developed a reversible method of tetracycline-dependent transcriptional suppressor-induced downregulation of 5-HT1A autoreceptors, but not 5-HT1A heteroreceptors. Mice induced to show lower levels of 5-HT1A presynaptic autoreceptors showed a markedly improved response to fluoxetine (SSRI) treatment in multiple depressive tests. Additionally, extracellular 5-HT concentrations 8 d after SSRI treatment were significantly higher in 1A^+/−^ mice when compared with 1A^+/+^ mice. Experiments using irreversible 5-HT1A knock-out mice have also reported a favorable antidepressant response in mice with reduced expression of 5-HT1A autoreceptors (Bortolozzi et al., 2012; Ferrés-Coy et al., 2013). These studies imply that modifications to the 5-HT1A receptors could be beneficial in the treatment and remission of depression.

### Clinical implications

Many studies have reported that the administration of both 5-HT1A antagonists (Blier and Ward, 2003; Berney et al., 2008) and agonists (Koek et al., 1998; Papakostas et al., 2004) result in antidepressant effects in treatment-resistant depressive patients. Weak antagonists, such as pindolol, reportedly bind to 5-HT1A receptors without causing a reduction in 5-HT firing rates, while preventing further inhibition and, consequently, desensitization from occurring (Albert, 2012). Agonists, however, are thought to work via 5-HT1A autoreceptor firing, 5-HT1A autoreceptor downregulation, and disinhibition of the 5-HT neuron, resulting in increased raphe 5-HT firing (Rotondo et al., 1997; Koek et al., 1998).

This 5-HT1A autoreceptor downregulation and raphe disinhibition, which can occur over a timespan of weeks, is thought to be one of the main pathways in which SSRIs mediate a reduction of depressive symptoms. It is also thought to account for the 4-8 week delayed response observed during SSRI treatment (Gartside et al., 1995; Blier, 2010; Richardson-Jones et al., 2010). As extracellular levels of 5-HT are increased in the synapse due to blocked 5-HTT, 5-HT1A receptors initially become desensitized within minutes of activation (Ferguson, 2001; Shenoy and Lefkowitz, 2003). Consistent ligand binding of the 5-HT1A somatodendritic autoreceptors leads to compensatory downregulation of 5-HT1A autoreceptors and reduced gene expression, causing disinhibition of the raphe cells (Albert and Lemonde, 2004). This disinhibition has been shown to lead to increased raphe firing and higher extracellular 5-HT concentrations in the weeks following SSRI treatment (Richardson-Jones et al., 2010). As this is the case, some studies have shown that augmenting SSRI treatment with 5-HT agonists, or antagonists, helped to achieve a greater reduction in depressive symptoms in patients with treatment-resistant depression (Trivedi et al., 2006; Berney et al., 2008). Because both the 5-HT1A and 5-HTT genes have been implicated in response to antidepressant treatment, it is imperative that future research accounts for both genes, and that future treatments incorporate agents that act on both proteins.

## 5-HT1A and 5-HTTLPR combined effect

### Grounds for a relevant model

Because antidepressant augmentations that impinge upon 5-HT1A receptors have been shown to be an effective form of treatment for depression, polymorphisms within this region have become a topic of interest during the past decade (Suzuki et al., 2004; Drago et al., 2008). The previously mentioned C(-1019)G polymorphism is one of the most widely studied mutations in the 5-HT1A receptor, and many studies (Hong et al., 2006; Malaguti et al., 2011) have shown that the 1A^G/G^ genotype mediates an attenuated response to antidepressant treatment, although it should be noted that not all studies have found an association with this polymorphism and antidepressant response (Xu et al., 2012). Similarly, while several studies have demonstrated that the 5-HTTLPR polymorphism moderates the response to SSRI treatment (Smeraldi et al., 1998; Rausch et al., 2002; Huezo-Diaz et al., 2009), there are still multiple studies that have reported no association between the 5-HTTLPR genotype and response to SSRIs (Kraft et al., 2007; Dogan et al., 2008; Wilkie et al., 2008). There are many factors that could lead to the discrepancies found among these studies, which have been reviewed elsewhere (Serretti et al., 2007; Porcelli et al., 2012). However, it is possible that other polymorphisms, not accounted for, could cause there to be no observable difference in SSRI response. Both polymorphisms have a high population prevalence in Caucasians [5-HTT^L/L^ = 0.331, 5-HTT^L/S^ = 0.474, 5-HTT^S/S^ = 0.195 (Odgerel et al., 2013); 1A^C^ = 0.675, 1A^G^ = 0.325 (Drago et al., 2008)]. Considering how both of these polymorphisms can alter the response to SSRI treatments, it is highly possible that these unmeasured genotypes have been a confounding factor in previous studies.

A search of the relevant literature yielded only two studies (Arias et al., 2005; Hong et al., 2006) that have examined the relationship between both polymorphisms and the response to SSRI treatment. In both studies, a combined effect was reported. Arias et al. (2005) observed that participants with the 5-HTT^S/S^–1A^G/G^ genotype (each independently shown to be the least responsive to SSRI treatment) reported a significantly less favorable response to SSRI administration than any other genotype. Conversely, Hong et al. (2006) found that of all the genotypes tested for SSRI response, patients with the 5-HTT^L/L^–1A^C/C^ genotype (each independently shown to be the most responsive to SSRI treatment) responded significantly better than participants with any of the other genotypes. These results, along with research that associates the 5-HTT and 5-HT1A polymorphisms with a combined increased susceptibility to depression (Zhang et al., 2009), demonstrate that a more nuanced approach is necessary for determining the relationship between SSRI response and genotype. While these studies did report a gene–gene response to SSRI treatment, no neural model was offered as to why these effects were observed. Additionally, no mechanism, to date, has been proposed as to why these genotypes show a combined response to antidepressant treatment.

### Model

Based on the knowledge currently available about the two polymorphisms, a model is presented that might account for the gene–gene interaction effect and provide a framework to guide future work in this area. As noted above, 5-HTT^+/−^ mice with reduced amounts of expressed 5-HTT, analogous to the 5-HTT^S/S^ genotype in humans, owing to their reduced expression of 5-HTT (Murphy et al., 2001; Murphy and Lesch, 2008; Kalueff et al., 2010), show no discernible differences in extracellular 5-HT concentration when compared with 5-HTT^+/+^ mice (analogous to the 5-HTT^L/L^ genotype in humans; Mathews et al., 2004). Following SSRI treatment, increases in extracellular 5-HT concentrations were larger in 5-HTT^+/+^ mice than increases in extracellular concentrations observed in 5-HTT^+/−^ mice (Shen et al., 2004).

**Figure 1:**
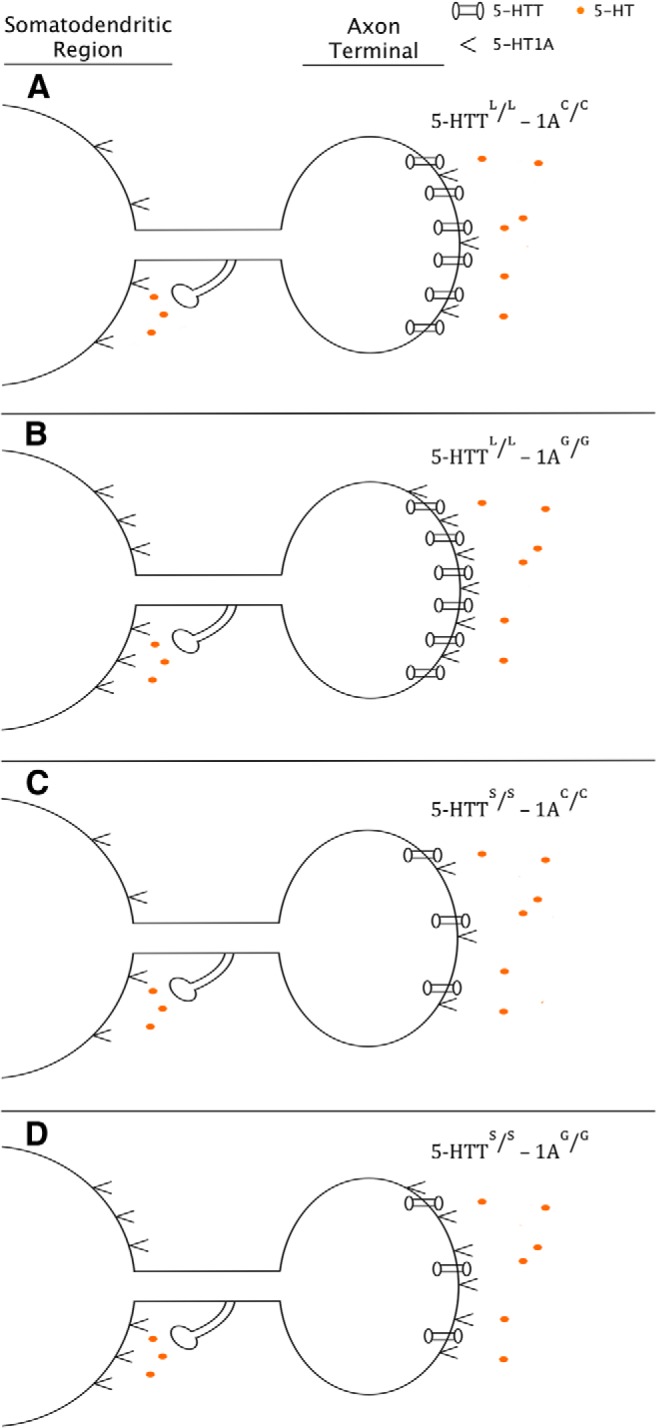
Pre-SSRI treatment. Diagram depicting extracellular 5-HT concentration, 5-HTT expression, and 5-HT1A receptor expression across the 5-HTTLPR and 5-HTR1A genotypes. ***A***, ***B***, Increased expression of 5-HTT due to the 5-HTT^L/L^ genotype. ***C***, ***D***, Decreased expression of 5-HTT due to the 5-HTT^S/S^ genotype. ***A***, ***C***, Reduced expression of 5-HT1A due to the 1A^C/C^ genotype. ***B***, ***D***, Reduced expression of 5-HT1A due to the 1A^G/G^ genotype. While studies have demonstrated that there are fewer 5-HT1A expressed receptors in individuals with the 5-HTT^S/S^ genotype (David et al., 2005), for the sake of simplicity in the model, no interaction is assumed between the two polymorphisms.

**Figure 2:**
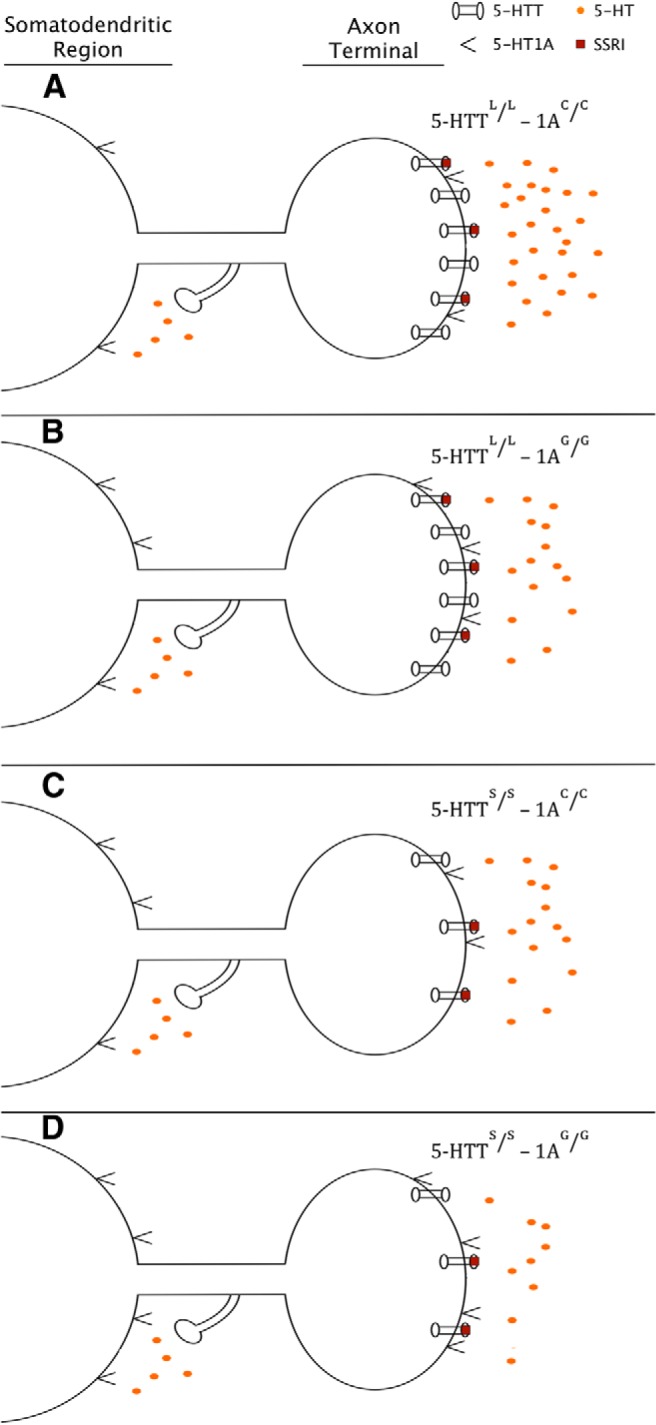
Post-SSRI treatment. Diagram depicting extracellular 5-HT concentration, 5-HTT expression, and 5-HT1A receptor expression across the 5-HTTLPR and 5-HTR1A genotypes after long-term SSRI treatment. Subsequent to somatodendritic downregulation of 5-HT1A autoreceptors, disinhibition of raphe neurons causes an increase in 5-HT release. ***A***-***C***, However, genotypes that express fewer 5-HTTs or higher numbers of 5-HT1A receptors (***B***, ***C***) show a reduced increase in extracellular 5-HT concentration when compared with the genotype that has higher 5-HTT levels and lower numbers of 5-HT1A receptors (***A***). ***D***, The genotype expressing both reduced levels of 5-HTT and higher numbers of 5-HT1A receptors shows the smallest increase in extracellular levels of 5-HT.

In other murine models, 1A^+/+^ mice with a higher expression of 5-HT1A autoreceptors, analogous to the 1A^G/G^ genotype in humans, express higher 5-HT1A presynaptic binding and a weakened response to SSRI treatment when compared with the 1A^+/−^ genotype, which is analogous to the human 1A^C/C^ genotype (Richardson-Jones et al., 2010). This blunted response is attributed to larger amounts of presynaptic terminal inhibition due to increased 5-HT1A autoreceptor binding (Parsey et al., 2010). Additionally, after SSRI treatment and somatodendritic downregulation of 5-HT1A autoreceptors, 1A^+/−^ mice initially expressing lower numbers of 5-HT1A autoreceptors had markedly higher extracellular 5-HT concentrations than that of their 1A^+/+^ littermates (Richardson-Jones et al., 2010). These results together form the basis for the proposed model, which describes a genotype-dependent modulation of postsynaptic serotonin signaling associated with the 5-HTT and 5HTR1A genes (Figs. 1, 2).

#### 5-HT^L/L^–1A^C/C^ genotype

While there would be no marked difference in initial extracellular 5-HT levels between 5-HTTLPR genotypes, after SSRI treatment high amounts of extracellular 5-HT in the synapse would be present, specifically in contrast with individuals with the 5-HTT^S/S^ genotype. The high amounts of extracellular 5-HT would then bind to the low amounts of 5-HT1A autoreceptors (when compared with the 1A^G/G^ genotype) on the presynaptic cell and would lead to a mild-to-moderate amount of inhibition. After downregulation 5-HT1A autoreceptors after somatodendritic 5-HT1A receptor binding, larger amounts of serotonin would be released into the synapse when compared to the 1A^G/G^ genotype (as demonstrated by Richardson-Jones et al., 2010). Due to the high amounts of extracellular 5-HT from 5-HTT binding and increased 5-HT release from reduced presynaptic terminal inhibition, the largest increase in 5-HT levels would occur in these individuals. This genotype is hypothesized to result in the highest amount of postsynaptic 5-HT signaling and would show the most favorable response to SSRI treatment.

#### 5-HTT^L/L^–1A^G/G^ genotype

After SSRI treatment, individuals with this genotype would initially have the same increase in extracellular 5-HT levels as the 5-HTT^L/L^–1A^C/C^ genotype, but due to the higher number of 5-HT1A autoreceptors there would be an increased amount of inhibition in the presynaptic terminal, leading to low amounts of 5-HT released. After somatodendritic downregulation, only a moderate increase in 5-HT levels would be observed, owing to the high serotonin concentration from the blocked 5-HTT and the low amount of 5-HT released due to the high 5-HT1A autoreceptor density. This genotype is hypothesized to result in a moderate amount of postsynaptic 5-HT signaling and would show an intermediate response to SSRI treatment.

#### 5-HTT^S/S^–1A^C/C^ genotype

After SSRI treatment, individuals with the 5-HT^S/S^ genotype would have a small increase in extracellular 5-HT concentrations when compared with the 5-HTT^L/L^ genotype. This small amount of 5-HT binding to the low number of 5-HT1A autoreceptors would lead to a smaller magnitude in the downregulation of the somatodendritic cell, when compared with downregulation from the 5-HT^L/L^ genotypes. This reduced amount of downregulation, and consequently the low amounts of disinhibition, would be offset, however, by the reduced expression of presynaptic 5-HT1A autoreceptors already present due to the 1A^C/C^ genotype, resulting in a moderate increase in 5-HT levels. This genotype is hypothesized to result in a moderate level of postsynaptic 5-HT signaling and show an intermediate response to SSRI treatment (possibly similar to that of the 5-HTT^L/L^–1A^G/G^ genotype).

#### 5-HTT^S/S^–1A^G/G^ genotype

Following SSRI treatment, individuals with the 5-HTT^S/S^–1A^G/G^ genotype would have the same increase in extracellular 5-HT concentrations as the genotype 5-HTT^S/S^–5-HTT^C/C^, but, due to the increased number of 5-HT1A autoreceptors present, a higher amount of presynaptic inhibition would occur. In addition, once downregulation of the somatodendritic 5-HT1A receptors occurred (similar to that of the 5-HTT^S/S^–1A^C/C^ genotype), less 5-HT would be released into the synapse due to the higher density of the 5-HT1A autoreceptors inhibiting the presynaptic terminal. The small amount of extracellular serotonin from 5-HTT binding, combined with the small amounts of 5-HT being released due to high 5-HT1A autoreceptor density, would lead to the smallest increase in 5-HT levels. This genotype is hypothesized to result in the least amount of postsynaptic 5-HT signaling and would show the least favorable response to SSRI treatment.

## Discussion

The proposed model predicts that the 5-HTT^L/L^–1A^C/C^ genotype will produce the highest amount of postsynaptic 5-HT signaling, that the 5-HTT^L/L^–1A^G/G^ and 5-HTT^S/S^–1A^C/C^ genotypes will produce a moderate amount of postsynaptic 5-HT signaling, and that the 5-HTT^S/S^–1A^G/G^ genotype will produce the least amount of postsynaptic 5-HT signaling. In addition to predicting which individuals will respond more favorably to antidepressant treatments, this model can also be used to predict the temporal dynamics associated with response to treatment. More specifically, while the functional outcomes of the 5-HTT^L/L^–1A^G/G^ and 5-HTT^S/S^–1A^C/C^ genotypes are predicted to be similar, these genotypes may behave differently before they reach equilibrium. Because the 5-HTT^L/L^–1A^G/G^ genotype contains a high density of inhibitory 5-HT1A autoreceptors, the benefits of the SSRI treatment will be observed only after the autoreceptors have been downregulated. Only after the autoreceptors have been downregulated will the increase in extracellular 5-HT levels be detectable. In contrast, the 5-HTT^S/S^–1A^C/C^ genotype has a significantly lower density of inhibitory 5-HT1A autoreceptors. It is possible that the main benefits of SSRI treatment could be observed earlier than the 5-HTT^L/L^–1A^G/G^ genotype, as the model predicts that the 5-HTT^S/S^–1A^C/C^ genotype relies less on somatodendritic downregulation of 5-HT1A autoreceptors and more on the initial inhibition of 5-HT reuptake. Future research can examine whether genotype moderates the temporal response to antidepressant treatment, as well as the end response.

Ultimately, though, the model predicts the response to SSRI treatment based on genotypic modulation of postsynaptic 5-HT signaling. This genotype-dependent modulation of postsynaptic serotonin signaling is important, as higher postsynaptic serotonin signaling is associated with reduced depressive symptoms. This is, in part, thought to be due to the triggering of chemical cascades that ultimately results in both increased dendritic arborization and brain-derived neurotrophic factor (BDNF) expression within hippocampal cells (Nestler et al., 2002). Increased 5-HT signaling via SSRI treatment has been reported to enhance BDNF expression (Duan et al., 2004), which is associated with a reduction in depressive symptoms (Martinowich and Lu, 2008). It is because of these downstream pathways that 5-HT levels are implicated in both susceptibility to and treatment of depression, and why SSRIs, which increase postsynaptic 5-HT signaling, are effective in treating depression. This also explains why participants with the 5-HT^L/L^–1A^C/C^ genotype respond more favorably to SSRI treatment than other genotypes and why individuals with the 5-HTT^S/S^–1A^C/C^ genotype have poorer treatment response than those with other genotypes (Arias et al., 2005; Hong et al., 2006), as the modulated 5-HT increases associated with each genotype would alter induced BDNF expression.

Further research should be conducted to determine whether differences in BDNF expression, and other downstream responses associated with 5-HT signaling, are observed between individuals with differing genotypes, as this would add further support for the proposed model. Additionally, researchers have recently developed a 5-HT1A agonist (F15599) specifically targeted for 5-HT1A heteroreceptors (Lladó-Pelfort et al., 2010). Individuals with a blunted response to SSRI treatment due to genotype could benefit from 5-HT1A heteroreceptor agonist augmentation as it could compensate for the marginal increase in extracellular 5-HT available for postsynaptic signaling.

While this model proposes an explanation for the gene–gene interaction observed by the two previously mentioned studies (Arias et al., 2005; Hong et al., 2006), it is insufficient to explain why some patients fail to respond to antidepressant treatment, whereas others who have a genetic predisposition never actually succumb to depression. This is due to the highly plastic nature of the serotonin system, which can easily compensate for a disruption in one aspect of the 5-HT system (Holmes, 2008). Extensive research is still necessary before widespread use of genetic testing in tailoring antidepressant treatment to individuals can be implemented, and other gene–gene interactions (including gene–gene–gene and gene–gene–environment interactions) should be studied further. This model advances the current understanding of how genotype can influence the neuronal response to antidepressants and can help to guide future research on the topic of SSRI response.

## Synthesis

The decision was a result of the Reviewing Editor Karen Szumlinski and the peer reviewers coming together and discussing their recommendations until a consensus was reached. A fact-based synthesis statement explaining their decision and outlining what is needed to prepare a revision is listed below. The following reviewers agreed to reveal their identity: Skirmantas Janusonis, Christian Muller

The revised theoretical review, entitled "Genetic variations in the serotonergic system mediate a combined, weakened response to SSRI treatment: a proposed model" was deemed acceptable with minor modifications (mainly grammatical errors and typos), following consultation with the 2 original reviewers of the paper. The reviewers and I all think that the manuscript is much improved and the model more easy to follow and we all appreciate your efforts in addressing their concerns. I concur with the assessments of this report by our expert reviewers and agree that, once the list of typos and errors is corrected (as outlined by Reviewer 1), the manuscript will be acceptable for publication in eNeuro.

### Reviewer 1:

The authors have considerably improved the manuscript. The model is stated early (p. 6), which makes the following statements much easier to follow. However, the manuscript still contains some errors and typos:

1. Abstract, lines 4-7: "Similarly, individuals with the C(-1019)G polymorphism in the promoter region of the serotonin 1A receptor gene (5-HTR1A) have shown blunted responses to SSRI treatment when compared with individuals lacking this polymorphism." A polymorphism is a population measure and is not applicable to individuals. Individuals can have only specific alleles of a polymorphism.
2. Page 5, lines 99-100: "[...] the transcription rate of both the 5-HTT and the 5-HT1A receptor is altered when these polymorphisms are present [...]" See my previous comment.
3. Page 7, lines 156-159: "As the 5-HTT(S) allele is associated with a reduction in the transcription rate of the 5-HTT by a factor of two, neurons with the 5-HTT(s) allele express half the amount of 5-HTT when compared with neurons with the 5-HTT(L/L) genotype." The second "s" should be capitalized. Also, this seems to refer to the presence of only one "S" allele (as opposed to 5-HTT(S/S)). Please clarify it.
4. Page 8, line 170: "Following SSRI treatment if the same proportion [...]" It's understandable but awkward.
5. Page 8, line 182: "Mainly because individuals [...]" This sentence appears incomplete.
6. Page 9, lines 187-188: The "S" and "L" should be superscripted.
7. Page 10, line 228: "[...] the human 5q11.2-13 chromosome [...]. There is no such chromosome. It's a locus on chromosome 5, which can be referred to as "chromosome 5q11.2-13."
8. Page 11, lines 233-234: "Because Pet-1 is only expressed in raphe neurons [...]" Raphe neurons or brain serotonergic neurons (that are in the raphe nuclei)?
9. Page 11, line 239: "Freud1/2" is misspelled; also, the authors may want to consider "Freud-1/2" (which would also be more consistent with "Pet-1").
10. Page 11, line 240: "[...] in the brain and the nervous system [...]". In the central nervous system? Or in the central and peripheral nervous systems?
